# Detection of antimicrobial peptides from fecal samples of FMT donors using deep learning

**DOI:** 10.3389/fvets.2025.1689589

**Published:** 2025-10-14

**Authors:** Songlin Wei, Huifang Yin, Xinliang Hu, Yulang Chi, Lishan Zhang, Bangzhou Zhang, Kai Qian, Wei Xu

**Affiliations:** ^1^School of Information Engineering, Xiamen Ocean Vocational College, Xiamen, China; ^2^Xiamen Treatgut Biotechnology Co., Ltd., Xiamen, China; ^3^College of Oceanology and Food Science, Quanzhou Normal University, Quanzhou, China; ^4^Basic Medicine College, Yichun University, Yichun, China

**Keywords:** antimicrobial peptides, fecal microbiota transplantation, fecal metagenome, deep learning, molecular dynamics simulations

## Abstract

**Introduction:**

Antimicrobial peptides (AMPs) represent a class of short peptides that are widely distributed in organisms and are regarded as an effective means to tackle bacterial resistance, potentially functioning as substitutes for onventional antibiotics.

**Methods:**

We employed metagenomics in combination with deep learning to mine AMPs from the 120 fecal microbiota transplantation (FMT) donor metagenome. Subsequently, a comprehensive analysis of the candidate AMPs was conducted through metaproteomic cross-validation, solubility analysis, cross-validation with other prediction tools, correlation analysis, and molecular dynamics simulations. Finally, four candidate AMPs were selected for chemical synthesis, and experimental validation identified two with broad-spectrum antimicrobial activity. Furthermore, molecular docking was utilized to further analyze the antimicrobial mechanisms of the candidate AMPs.

**Results:**

Our approach successfully predicted 2,820,488 potential AMPs. After a comprehensive analysis, four candidate AMPs were selected for synthesis, two of which exhibited broad-spectrum antimicrobial activity. Molecular docking provided further insight into the binding mechanisms of these peptides.

**Discussion:**

This study demonstrates the feasibility of discovering functional AMPs from the human fecal microbiome using computational and experimental approaches, highlights the potential of mining novel AMPs from the fecal microbiome, and provides new insights into the therapeutic mechanisms of FMT.

## 1 Introduction

Antibiotics represent one of the most significant discoveries in human history, having saved innumerable lives. Nevertheless, the overuse of antibiotics has led to a sharp rise in antibiotic-resistant bacteria, resulting in a significant annual death toll due to drug-resistant infections. *Escherichia coli* is accountable for the highest number of deaths, succeeded by *Klebsiella pneumoniae*, *Staphylococcus aureus*, *Acinetobacter baumannii*, *Streptococcus pneumoniae*, and *Mycobacterium tuberculosis* (1). This problem is intensifying progressively, and it is projected that by 2050, the number of deaths attributed to antimicrobial resistance could ascend to 1.51 million worldwide (2). To alleviate the issue of antibiotic resistance, alternative approaches to conventional antibiotic treatments have emerged. The current major alternative therapeutic strategies encompass antimicrobial peptides (AMPs), antibody-antibiotic conjugates (AACs), phage therapy, and microbiome-based therapies (3). Among these, AMPs have attracted extensive attention due to their potent antibacterial activity and low probability of developing resistance.

AMPs, also referred to as host defense peptides, are typically constituted by 2 to 50 amino acids. These small molecule peptides have the ability to inhibit bacteria, fungi, viruses, and other pathogens, and constitute an essential part of the innate immune system. AMPs are extensively distributed among animals, plants, and microorganisms (4). The structures of AMPs are diverse, with the main recognized types being (i) *α*-helical, (ii) *β*-sheet, (iii) αβ, or (iv) non-αβ elements (5). AMPs demonstrate bactericidal activity against the majority of major Gram-positive and Gram-negative bacteria. Their mechanisms of action are generally considered to encompass direct killing of bacteria by disrupting the bacterial cell membrane, as well as targeting crucial intracellular biological processes such as nucleic acid synthesis, cell wall biosynthesis, and enzyme production (6). Due to their diverse mechanisms of action, it is difficult for microorganisms to develop resistance to AMPs, making them one of the most promising candidates for antimicrobial drugs.

Early research on antimicrobial peptides (AMPs) primarily relied on laborious methods for extraction, isolation, purification, and functional validation from specific organisms or their metabolic products (7). For instance, the discovery of cecropin (8) and magainin (9) laid the foundation for understanding AMPs as key effector molecules in innate immunity. With advances in genomics and proteomics, high-throughput identification of AMP families has been achieved through homology cloning and sequence mining (10, 11). In recent years, the integration of high-throughput sequencing technologies, artificial intelligence, and molecular dynamics simulations has significantly enhanced the efficiency of AMP development. The Ma team combined LSTM, Attention, and BERT models to identify 2,349 candidate AMPs from human gut microbiome data, synthesized 216 of them, and discovered that 181 displayed antimicrobial activity (83% positivity rate) (12). The Huws group employed a classifier model to identify two AMPs effective against multidrug-resistant (MDR) bacteria from a rumen microbial metagenomic dataset (13). The Liang team established a structure–activity relationship-based virtual screening platform to screen 3.44 million peptides from the UniProt database and verified the top three scoring peptides (14). The Fuente-Nunez team employed the deep learning framework APEX 1.1 to conduct a systematic, large-scale screening for antibiotics within archaeal proteomes. This effort successfully predicted and identified a novel family of antimicrobial peptides named “archaeasins” (15).

**Figure 1:**
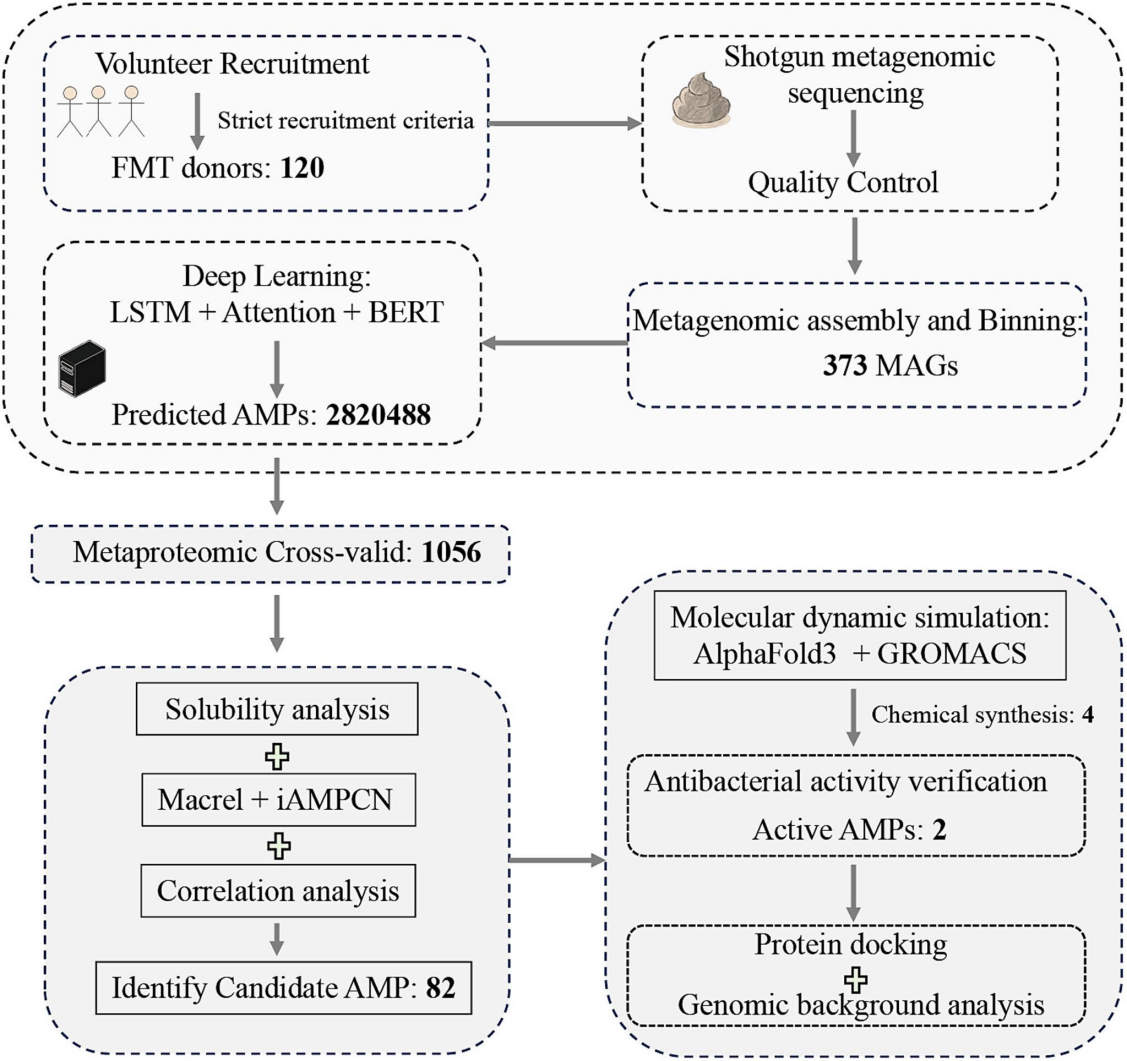
Schematic workflow of the integrated computational and experimental approach for AMPs discovery from FMT donor samples.

The human gastrointestinal tract constitutes a vast microbial ecosystem, hosting trillions of microorganisms. Its gut microbiome encodes an extremely diverse set of genes. Research suggests that a considerable number of AMP families within the human gut microbiome have yet to be comprehensively investigated (12, 16). Hence, in this study, we employed fecal metagenomic samples from donors who conformed to the consensus criteria for Fecal Microbiota Transplantation (FMT) to search for candidate AMPs (17). FMT, involving the utilization of microorganisms derived from human feces to reestablish the gut microbiota, has a rigorous donor selection process. As a therapeutic approach for human diseases, FMT is utilized to treat *Clostridium difficile* infection, multidrug-resistant organism infections, and other disorders (18). We extended the existing methods for AMP development by searching for candidate AMPs based on fecal metagenomic samples. We explored an efficient workflow for AMP development (Figure 1), and identified AMPs with broad-spectrum antimicrobial activity. Additionally, we conducted in-depth analyses of the identified candidate AMP sequences.

## 2 Materials and methods

### 2.1 Sample collection and metagenomic sequencing

The recruitment of FMT donors undergoes a rigorous process, which is divided into four stages: initial questionnaire screening, on-site interview, comprehensive physical examination, and medical verification. The primary characteristics of the donors are as follows: good health, absence of infectious diseases or pathogens that could be transmitted to recipients. They are also required to maintain regular dietary and sleep habits and avoid unhealthy lifestyles. Furthermore, the intestinal microbiota should demonstrate high diversity and stability, with no recent use of antibiotics. Finally, a total of 120 fecal samples fulfilling the FMT donor criteria were gathered from Jiangxi Province, China. The sample collection process was designed to guarantee the freshness and non-contamination of the samples. During the collection, a sterile disposable plastic spatula was employed to collect substances from the surface, interior, and middle sections of the stool, which were subsequently placed in sterile and sealed containers to prevent contamination with urine or disinfectants. Each sample weighed approximately 0.25–0.5 g and was promptly cooled to −20 °C or −80 °C after aliquoting to avoid repeated freezing and thawing. Alternatively, samples were stored at 4 °C and transported to the laboratory within 2 weeks. The entire collection procedure was carried out under rigorous sterile conditions to ensure sample quality, rendering them suitable for subsequent metagenomic sequencing analysis. Bacterial genomic DNA was extracted from the fecal samples using a fecal bacterial DNA extraction kit. Shotgun sequencing was conducted on the Illumina platform with an insert size of 150 bp.

### 2.2 Metagenomic analysis

The raw sequencing data were initially subjected to quality assessment via FastQC.^1^ Subsequently, low-quality bases and sequencing adapters were eliminated using Fastp (19). KneadData was employed to eliminate host genome contamination. The metagenomic sequences were rapidly assembled by using Megahit (v1.2.9) (with parameter setting: --k-min 29 --min-contig-len 1,000) (20), and the assembly quality was evaluated using QUAST (v5.0.2) (21). Subsequently, the MetaWRAP (22) was utilized for the binning step, and the CheckM (23) tool was employed to assess the completeness and contamination of the bins. To improve the efficiency of downstream analyses, the dRep (24) was applied to eliminate bin redundancy. For obtaining non-redundant bin taxonomic information, the CAT_pack (25) tool was utilized for bin classification and annotation, making use of GTDB data for rapid annotation. Functional annotation of the bins was also conducted using the MetaWRAP.

### 2.3 Antimicrobial peptides prediction

A deep learning model developed by Ma et al. (12) was employed for the prediction of AMPs. The method employs a combination of natural language processing (NLP) models, including LSTM, Attention and BERT, to create a unified computational pipeline that effectively identifies AMPs from human gut microbiome data by learning deep sequence features rather than relying on sequence similarity. A key strength of this approach is its high precision (91.31%) and low false-positive rate, enabling the discovery of novel AMPs with low homology to known sequences (<40% identity), of which 181 out of 216 synthesized peptides exhibited antimicrobial activity (>83% positive rate). Despite its success, the method depends heavily on the quality of existing training data, requires substantial computational resources, and leaves the mechanism of some effective peptides unclear; nevertheless, it demonstrates significant potential for accelerating the discovery of AMP candidate molecules using machine learning and large-scale metagenomic datasets.

Small open reading frames (sORFs) were derived from the bin genomes by applying the ‘getorf (−find 2 -table 11 -minsize 15 -maxsize 150)’ command in EMBOSS (version 6.6.0.0) (26). A Perl script was employed to eliminate redundancy and known AMP sequences, followed by the prediction of candidate AMPs through the established pipeline.

### 2.4 Metaproteomic cross-validation

To guarantee the expressibility of the predicted sORFs, we further conducted cross-validation by employing metaproteomic data. The data obtained from Herold et al. underwent additional filtering to acquire a non-redundant protein dataset with sequences shorter than 50 amino acids. Subsequently, we computed the k-mers of the sORFs and compared them with the metaproteomic dataset. If a k-mer was consistent with a peptide sequence in the metaproteomic data, it signified that more than half of the sORF existed as a peptide, indicating that the sORF had a higher probability of being expressed.

### 2.5 Correlation analysis

To establish a correlation network between candidate AMPs and bacteria, we initially computed the relative abundance of both the candidate AMPs and bacterial species in the metagenomic samples. We retrieved metagenomic samples from 100 healthy individuals from public databases, and subsequently conducted quality control using FastQC and Fastp. The relative abundance of bacterial species was acquired through Metaphlan 4 (27), an efficient species annotation tool based on marker genes.

To acquire the abundant information of AMPs, we aligned the candidate AMP sequences to the metagenomic reads by employing the PALADIN tool (28). Subsequently, the alignment results were processed using SAMtools (29) to compute the abundance. Only those AMPs with a prevalence of at least 5% and corresponding species abundance information were retained. The Spearman correlation between AMPs and bacterial species was calculated using the R package WGCNA (30), and the *p*-values were adjusted with the R package multtest.

### 2.6 Peptide selection

The R package Peptides was employed for the physicochemical property analysis of the predicted AMPs. The predicted AMP sequences were further cross-validated by means of two third-party tools, namely iAMPCN and Macrel, which adopt distinct techniques and strategies to identify and predict the functional activity of AMPs. iAMPCN is a deep learning model based on convolutional neural networks (31), while Macrel introduces a novel set of 22 peptide features designed to capture the physicochemical properties, structural characteristics, and sequence order information of AMPs (32).

To guarantee the excellent solubility of the synthesized AMPs, we further filtered the AMP sequences in accordance with six solubility evaluation criteria (33), retaining merely those sequences that met at least three of the criteria. The relevant analyses were conducted using a local R script. T-coffee was employed for the multiple sequence alignment of the candidate AMPs, and the resultant alignment file was utilized to construct a Neighbor-Joining phylogenetic tree with MEGA11 (34). Subsequently, the phylogenetic tree was visualized by means of iTOL (35).

We conducted molecular dynamics (MD) simulations on the candidate AMPs to evaluate their stability. Firstly, the three-dimensional structures of the candidate AMPs were predicted using AlphaFold3 (36), and the protein CIF files were obtained. Subsequently, the MD simulations were executed using GROMACS (37), with the AMBER99SB force field and the TIP3P water model. The simulation was carried out for 100 ns, and at the conclusion of the simulation, the stability of the AMPs was analyzed based on the root-mean-square deviation (RMSD). Finally, the candidate AMP for chemical synthesis is determined.

### 2.7 Peptide synthesis

The peptides investigated in this study were synthesized by GL Biochem Ltd. (Shanghai, China) via a solid-phase peptide synthesis strategy (SPPS). The accurate molecular weights were characterized by mass spectrometry. The purity of all peptides was determined using high-performance liquid chromatography, and all peptides exhibited a purity greater than 95%.

### 2.8 Bacteria strains and growth conditions

*E. coli* CICC 10667, *Pseudomonas aeruginosa* JCM5962 and *S. aureus* ATCC6538 were aerobically cultured at 37 °C in Luria-Bertani (LB) medium. *Staphylococcus epidermidis* CMCC26069, *Streptococcus mutans* ATCC 25175, *Propionibacterium acnes* ATCC 6919, and *Enterococcus faecalis* ATCC19433 were anaerobically cultured at 37 °C in Brain Heart Infusion Broth (BHI) medium.

### 2.9 Minimum inhibitory concentration determination

The minimum inhibitory concentrations (MICs) of the peptides were assessed following the method described by Chou et al. (38). Indicator bacteria cells were cultured at 37 °C in LB or BHI medium to log-phase growth and diluted to OD_600_ = 0.4, and then diluted 1,000-fold with fresh LB or BHI medium. In sterile 96-well plates, 50 μL of two-fold serial dilutions of AMPs in water, with predefined concentrations, were added to 50 μL of the diluted bacterial suspension. Subsequently, the plates were analyzed by means of a Microplate Reader at an optical density (OD) of 600 nm. The MIC was defined as the lowest concentration of the peptide that completely suppressed the visible growth of bacteria following 20 h of incubation at 37 °C. An AMP solution mixed with water served as the negative control, while a bacterial suspension combined with bacteria was utilized as the positive control.

### 2.10 Analysis of antibacterial mechanism

To explore the potential antibacterial mechanisms of the antimicrobial peptide, we initially retrieved the three-dimensional structural files of BamA, 1KZN, and 2XCT proteins from the Protein Data Bank (PDB) database. Subsequently, protein–protein interaction studies were conducted using GRAMM (Global RAnge Molecular Matching) with a free docking approach (39). The simulated docking results were assessed using the PDBePISA tool and visualized with PyMOL for structural representation.

### 2.11 Characteristic analysis of candidate AMP

To assess the distribution of cAMP573 within the *G. qucibialis* bacterial species, 69 genome files of this bacterial species were downloaded from NCBI database. Subsequently, sORFs were predicted using the ‘getorf’ command in EMBOSS. The parameter settings were configured as ‘-find 2 -table 11 -minisize 150’. The predicted sORFs were subjected to alignment with the sORFs of cAMP573 using the blastn tool. The parameter settings were configured as ‘-evalue 1e-10 -qcov_hsp_perc 90’. Subsequently, based on the alignment outcomes, the corresponding gene fragments were retrieved from the bacterial genome, with an extension of 10,000 base pairs on each side. Finally, the extracted gene fragments were annotated by means of the Prokka tool.

To conduct a comprehensive analysis of the candidate AMP cAMP314, 1,172 bacterial genome files were retrieved from the NCBI database. These files predominantly originated from species including *Phocaeicola vulgatus* (accounting for 48% of the total), *Phocaeicola dorei* (19%), and *Phocaeicola massiliensis* (4%). Subsequently, the analyses were carried out using the methods described above.

## 3 Result

### 3.1 Metagenomic binning and classification

**Figure 2:**
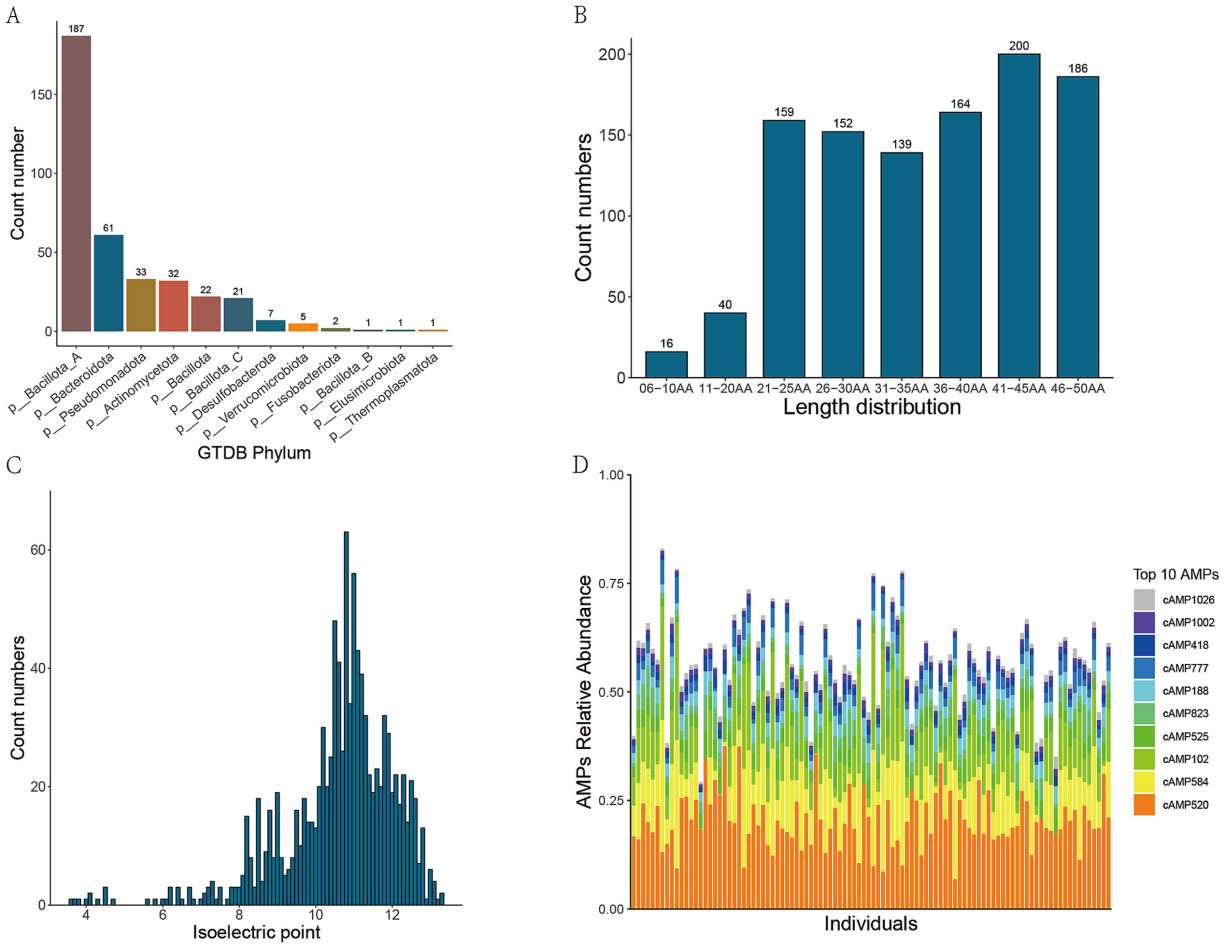
Metagenomic binning and species classification annotation results. **(A)**Phylum-level taxonomic distribution of the non-redundant, high-quality metagenome-assembled genomes (MAGs); **(B)** Bar chart depicting the length distribution of candidate AMPs through deep learning; **(C)** Distribution of isoelectric points of candidate AMPs; **(D)** The top 10 candidate AMPs by relative abundance in metagenomic samples from healthy individuals.

Following quality control, sequence assembly, binning, and redundancy elimination of 120 metagenomic sequencing datasets, a total of 373 non-redundant high-quality bins were successfully retrieved. To obtain the taxonomic information of the bins, classification annotation was carried out using the CAT_pack tool. The annotation findings indicated that, at the phylum level, 12 bacterial phyla were detected. Among them, *Bacillota* was the most prevalent, accounting for 187 bins, followed by *Bacteroidota* and *Pseudomonadota*, with 63 and 33 bins, respectively, (Figure 2A). At the genus level, 177 bacterial genera were identified. Among them, *Collinsella* and *Prevotella* had the highest number of bins, with 22 bins each, followed by *Haemophilus* and *Bacteroides*. At the species level, 187 bins were successfully annotated.

Following the processing of 373 bins via the ‘getorf’ command within EMBOSS, a cumulative total of 43,621,829 non-redundant sORFs were successfully retrieved. Subsequently, an integrated deep learning model was employed to predict AMPs from these sORFs, yielding 2,820,488 predicted AMPs. To enhance the probability that the predicted AMP sequences are expressible, cross-validation was conducted using metaproteomic data. After a series of filtering and other processing procedures, a final set of 1,056 candidate AMPs was meticulously identified. Among these AMPs, 5.3% exhibited a length of less than 20 amino acids, whereas 36.5% had a length exceeding 40 amino acids (Figure 2B). The isoelectric points of these peptides were predominantly concentrated within the range of 10 to 12 (Figure 2C).

Moreover, our findings indicated that the top 10 candidate AMPs with the highest relative abundances in the metagenomic samples from healthy individuals were predominantly derived from the following seven bacterial genera: *Copromonas* (cAMP520), *Gemmiger* (cAMP584 and cAMP102), *Ventrimonas* (cAMP525), *Eubacterium_I* (cAMP823), *Blautia_A* (cAMP188 and cAMP418), *Anaerobutyricum* (cAMP1026), and *Eubacterium_G* (cAMP1001) (Figure 2D).

### 3.2 Feature analysis of antimicrobial peptides

**Figure 3:**
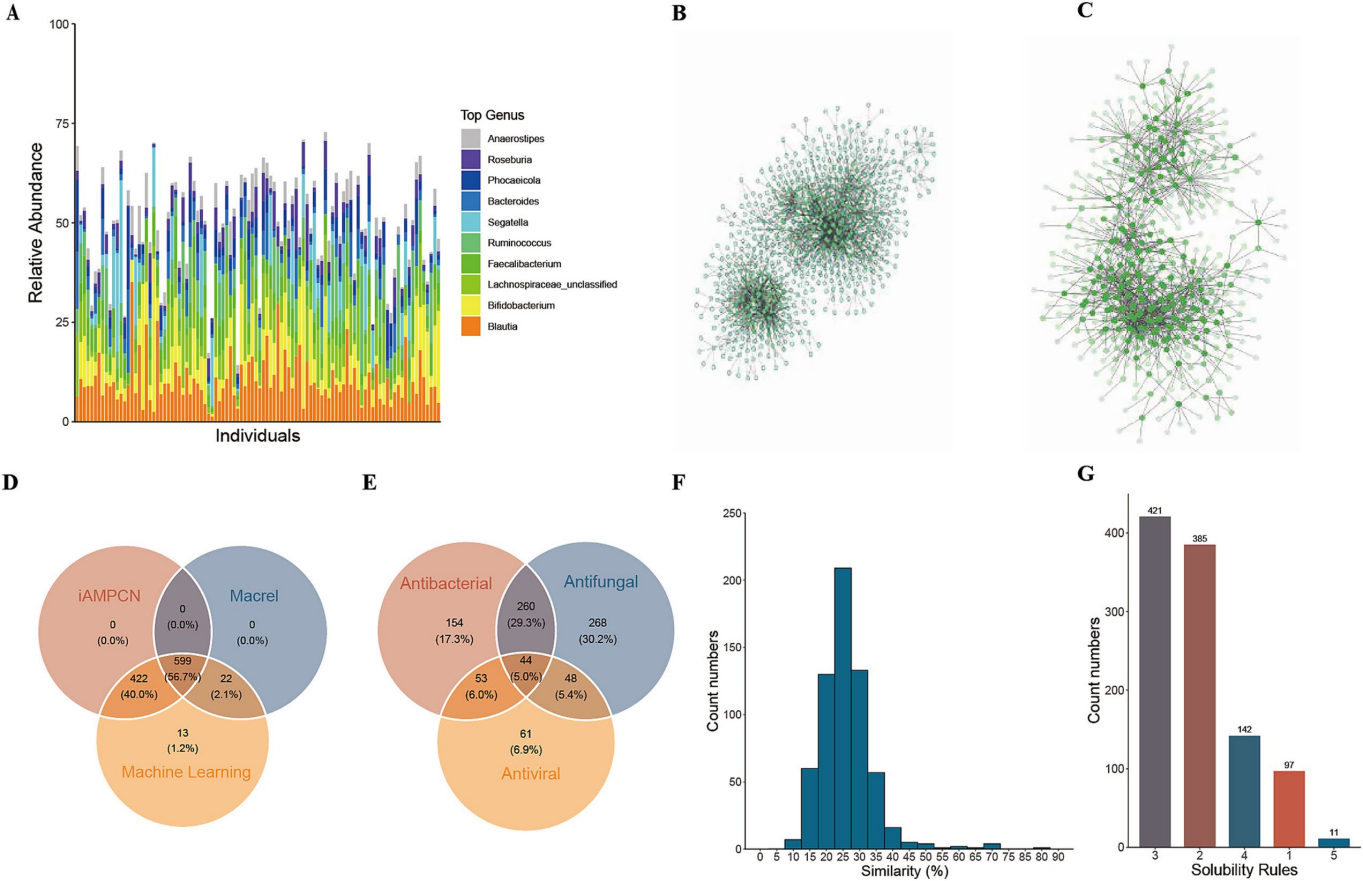
Correlation analysis results for enhancing the reliability of candidate antibacterial peptides. **(A)** Bar plot of the top 10 most abundant bacterial genera in the healthy cohort; **(B)** Negative interaction network between antibacterial peptides and bacteria at the species level in the healthy cohort; **(C)** Negative interaction network between antibacterial peptides and bacteria at the genus level in the healthy cohort; **(D)** The number of 1,056 candidate AMPs by Cross-validation results between two third-party tools, iAMPCN and Macrel; **(E)** Distribution of antibacterial peptides predicted by iAMPCN to have antibacterial, antifungal, and antiviral activities; **(F)** Similarity of candidate AMPs to antibacterial peptide sequences in the AMPSphere database; **(G)** Statistics of AMPs meeting different solubility evaluation criteria.

Given the known effects of some AMPs in regulating and stabilizing community structure (40, 41), Ma et al. (12) hypothesized that cAMPs with strong negative correlations with members of a microbiome thus potentially inhibit bacterial growth and are more likely to be functional, and this network could help further eliminate false positives in their discovery. Consequently, we endeavored to identify AMPs that display substantial negative interactions with bacteria via correlation analysis. The species annotation outcomes from Metaphlan4 revealed that the ten most abundant bacterial genera were *Blautia*, *Bifidobacterium*, *Lachnospiraceae*_unclassified, *Faecalibacterium*, *Ruminococcus*, *Segatella*, *Bacteroides*, *Phocaeicola*, *Roseburia*, and *Anaerostipes* (Figure 3A). Subsequently, through Spearman correlation analysis, we pinpointed 355 candidate AMPs that showed significant negative correlations with bacterial species (FDR < 0.05) (Figure 3B). At the genus level, 314 candidate AMPs were detected to have significant negative correlations with bacterial genera (FDR < 0.05) (Figure 3C).

To enhance the reliability of the predictions, cross-validation was conducted using two third-party tools, Macrel and iAMPCN. The analysis outcomes revealed that 1,021 candidate AMPs were predicted as AMPs by iAMPCN, while 621 candidate AMPs were predicted as AMPs by Macrel. Among them, 599 candidate AMPs were validated by both tools (Figure 3D). Moreover, the prediction results of iAMPCN demonstrated that 511 candidate AMPs might possess antibacterial activity, 660 could have antifungal activity, and 206 may exhibit antiviral activity. Additionally, 44 candidate AMPs were predicted to possess all three activities simultaneously (Figure 3E).

To evaluate the similarity between the predicted candidate AMP and AMP sequences in public databases, we downloaded all AMP sequences from the AMPSphere database as reference sequences. The candidate AMP sequences were then aligned to the database using the ‘needleall’ command in the EMBOSS software package. As a result, 630 candidate AMPs obtained valid alignment results. The analysis indicated that the candidate AMPs exhibited good novelty, with over 55% of the candidate AMP sequences showing less than 50% similarity to the reference sequences. The candidate AMP with the highest similarity was cAMP675 (82.6%) (Figure 3F).

In order to increase the probability of solubility of chemically synthesized cAMP, we further evaluated candidate AMP based on 6 protein solubility evaluation criteria. The analysis results showed that all candidate AMPs could not meet all 6 rules at the same time. Among them, 11 candidate AMPs met 5 rules, 142 met 4 rules, and 420 met 3 rules. That is, more than 54.26% of the candidate AMPs passed the solubility test (Figure 3G).

### 3.3 Select candidate AMP for chemical synthesis

**Figure 4:**
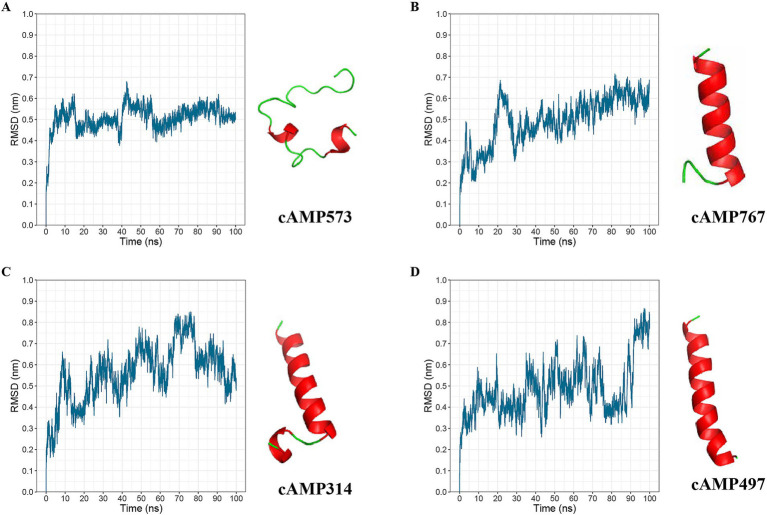
Molecular dynamics simulation results of AMPs. **(A)**, **(B)**, **(C)**, and **(D)** present the molecular dynamics simulation results of candidate AMPs cAMP573, cAMP767, cAMP314, and cAMP497, respectively. The left graphs show the molecular dynamics simulation results from GROMACS over a simulation time of 100 ns; the right side displays the predicted three-dimensional structures of the antibacterial peptides by AlphaFold 3.

To increase the probability that the synthesized AMPs would exhibit antimicrobial activity, we re-screened candidate AMPs based on the above analysis results. First, cross-validation of the 1,056 deep learning-predicted AMPs using a third-party tool retained 599 candidates. Second, selection of candidates showing negative correlations with bacterial species/genera retained 136 AMPs. Subsequently, application of solubility rules (≥3 criteria met) further refined the pool to 82 candidates. In addition, we performed molecular dynamics simulations on candidate AMPs using GROMACS and evaluated the stability of its structure using the RMSD value. Finally, we selected four candidate AMP sequences with lower RMSD values from the 82 candidate AMPs for chemical synthesis (Figure 4 and Table 1).

**Table 1:** The physicochemical characteristics and sequence of peptides.

Peptide name	Sequence	Relative abundance	Negatively correlated species	Length	Theoretical MW	Measured MW	Isoelectric point	Hydrophobicity
cAMP314	VKAFAGIEQVAQAIKAQKRCRFTFCG	2.83E-05	*Actinomyces; Eggerthella*	26	2871.41	2871.43	10.51	0.0088
cAMP497	VWKKLGKDFKVEFGLDVSERLCV	3.47E-04	*Guopingia; Wansuia*	23	2696.20	2696.22	8.53	−0.0022
cAMP573	RRWQPRPGWDNPVPAAKDTLTLPRWWWG	5.78E-04	*Mediterraneibacter; Anaerostipes*	28	3443.92	3443.92	12.05	−0.1821
cAMP767	LKTLLKSVVNRLGEAKGINA	1.44E-03	*Lachnoclostridium; Ruthenibacterium; Anaerotruncus; Massiliimalia;* *Guopingia*	20	2124.56	2124.57	11.08	0.021

### 3.4 Antibacterial activity evaluation of AMPs

We successfully synthesized 4 screened peptides by solid-phase peptide synthesis and tested the antibacterial activity of AMPs against *E. coli* CICC 10667, *P. aeruginosa* JCM5962 and *S. aureus* ATCC6538, *S. epidermidis* CMCC26069, *S. mutans* ATCC 25175, *P. acnes* ATCC 6919 and *E. faecalis* ATCC19433. Among them, two peptides (cAMP314 and cAMP573) exhibited broad-spectrum antimicrobial activity, inhibiting all seven selected indicator bacterial strains with MIC ranging from 32 to 256 μg/mL. However, the other two peptides were unable to completely inhibit the activity of the indicator bacteria (Table 2).

**Table 2:** Antimicrobial test results of synthetic peptides (MIC, μg/mL).

Peptide name	*E.coli*	*P.aeruginosa*	*S.aureus*	*S.epidermidis*	*S.mutans*	*P.acnes*	*E.faecalis*
cAMP314	32	256	64	64	32	32	256
cAMP497	>512	>512	>512	>512	>512	>512	>512
cAMP573	64	256	32	32	32	64	256
cAMP767	>512	>512	>512	>512	>512	>512	>512

### 3.5 Analysis of antibacterial mechanism

**Figure 5:**
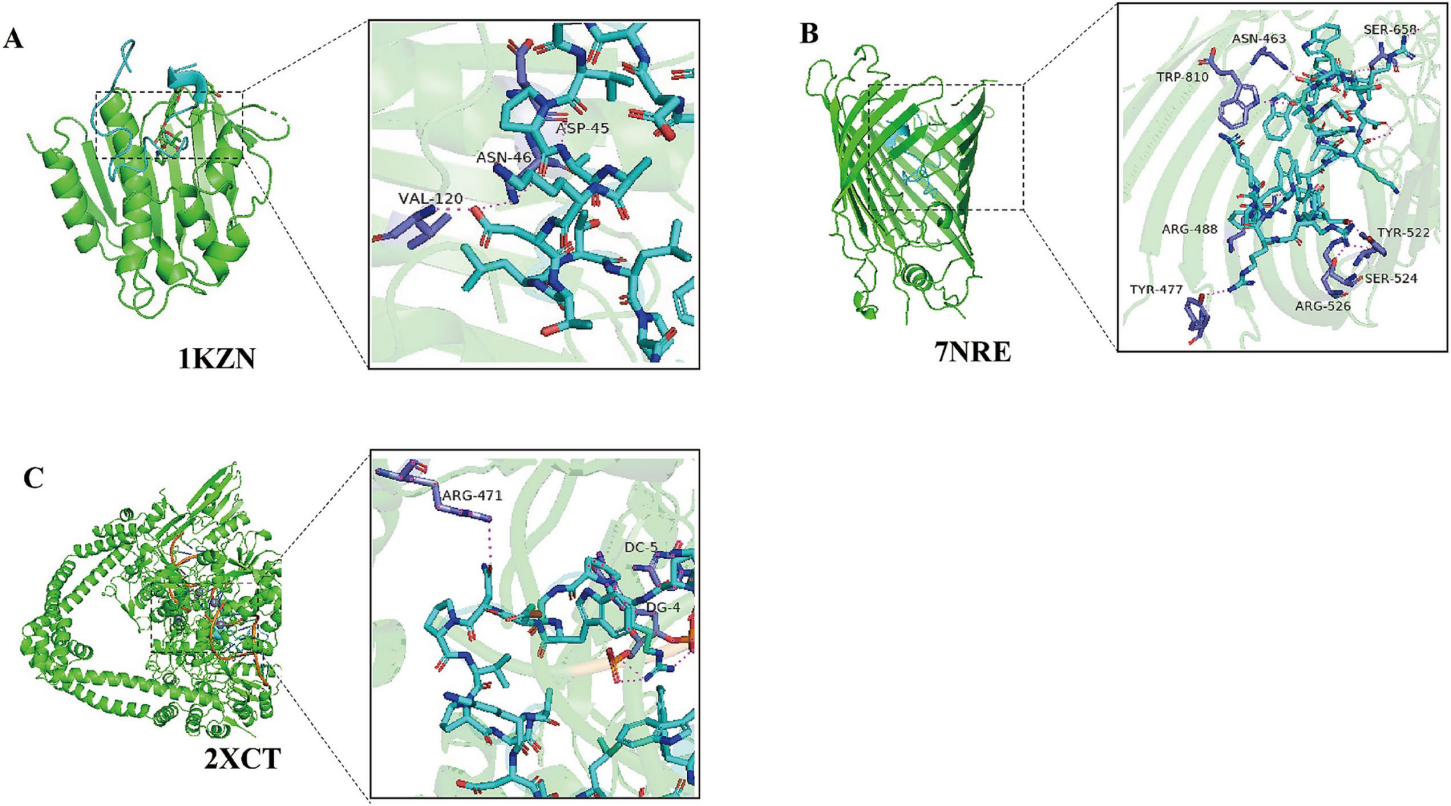
Simulated Docking Results between candidate AMP cAMP573 and Bacterial Proteins. **(A)** Simulated docking result between cAMP573 and protein 1KZN; **(B)** Simulated docking result between cAMP573 and protein 7NRE; **(C)** Simulated docking result between cAMP573 and protein 2XCT.

Protein docking simulation is an efficient method for studying molecular mechanisms of action. AMPs can inhibit bacteria by acting on cell membranes or intracellular enzymes. Therefore, we collected bacterial proteins from different sources during the protein docking simulation. DNA gyrase is essential for DNA synthesis, so we evaluated the interaction between cAMP573 and *E. coli* DNA gyrase 1KZN and *S. aureus* DNA gyrase 2XCT through protein docking simulation. At the same time, we also evaluated the interaction between cAMP573 and BamA protein, which is a membrane protein related to the virulence and antibiotic resistance of Gram-negative bacteria. Some AMPs can inhibit bacterial activity by binding to this protein (42). The simulation results show that the binding between cAMP573 and 2XCT protein is the most stable (ΔiG: −57.1 kcal/mol) (Figure 5C), followed by 1KZN protein (ΔiG: −5.6 kcal/mol) (Figure 5A). Although the binding free energy between cAMP573 and BamA protein (ΔiG: −3.6 kcal/mol) is greater than the −5 kcal/mol in the reference standard (43), the results still indicate that there may be a significant binding between the two (Figure 5B). cAMP314 exhibited a stable binding interaction with 2XCT (ΔiG: −18.5 kcal/mol), while displaying a relatively lower binding free energy with the BamA protein (ΔiG: −7.7 kcal/mol).

### 3.6 Characteristic analysis of cAMP573 and cAMP314

**Figure 6:**
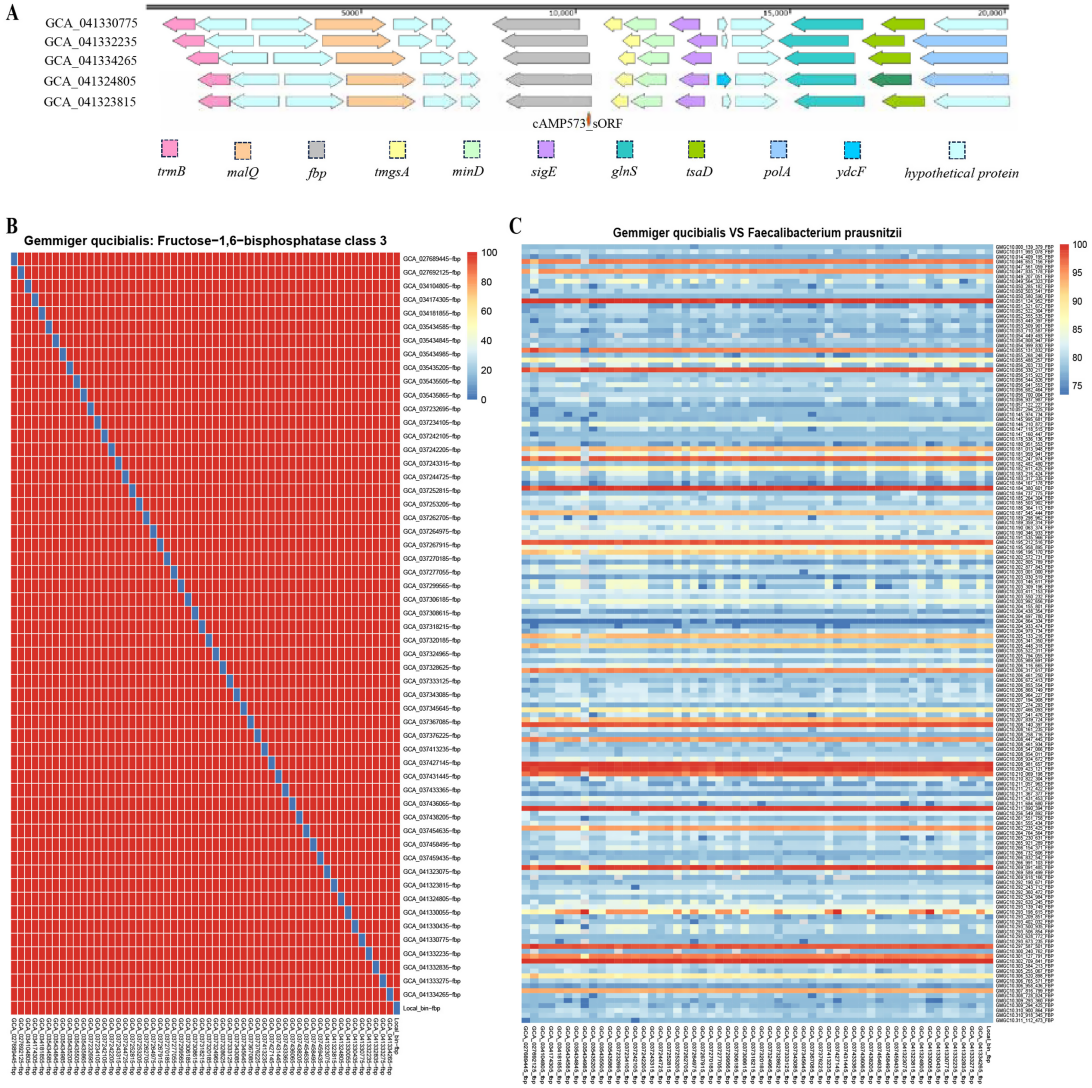
Genomic background information analysis results of sORFs encoding antibacterial peptides. **(A)** Genomic background information of the sORF encoding cAMP573; **(B)** Similarity analysis of the fbp gene across different *G. qucibialis* genomes; **(C)** Similarity analysis of the fbp gene between *G. qucibialis* and *F. prausnitzii* genomes.

The genomic bin containing cAMP573 was taxonomically annotated as *G. qucibialis.* Subsequent analysis revealed that the sORF of cAMP573 is widely distributed within *fructose-1,6-bisphosphatase class III* (*FBP-III*) genes across 55 genomes of this species. Moreover, the *FBP* gene is located within a specific genomic region of *G. qucibialis*, typically flanked upstream by *mgsA*, *minD*, and *sigE* genes, while downstream regions predominantly contain *trmB* and *malQ* genes (Figure 6A). By comparing the average nucleotide identity (ANI) of the *FBP-III* gene in different *G. qucibialis* genomes, we found that the genome was highly conserved among *G. qucibialis* bacterial species (Figure 6B). To evaluate the distribution of the sORF encoding cAMP573 in other bacterial species, we downloaded and aligned 19,391 *FBP-III* gene sequences from 81 bacterial species in the Global Microbial Gene Catalog (GMCC) database, but the presence of this sORF was not detected. Furthermore, analysis revealed that the FBP gene from *G. qucibialis* exhibited high similarity only with a limited number of sequences from *F. prausnitzii* (Figure 6C).

The sORF of cAMP314 is predominantly distributed within the *TonB* gene of *Phocaeicola massiliensis* in the *Phocaeicola* genus and genomes of multiple uncultured species. Further analysis revealed that the genomic region harboring this sORF exhibits high conservation across different genomes. The upstream region of the sORF was primarily flanked by a gene encoding a hypothetical protein and the *sigW* gene, while the downstream region contained conserved genes including *glaB* and *mggB* gene.

## 4 Discussion

The gut microbiome encodes highly diverse genes, being one of the largest reservoirs for antibiotic-resistant genes (44). At the same time, as a result of long-term competition and co-evolution, it is expected to produce a large number of antimicrobials against even multi-drug-resistant (MDR) bacteria (45). Research has shown that the human gut microbiome harbors a vast array of potential AMPs (12, 46, 47), which are anticipated to exhibit low toxicity, high stability, and mild antimicrobial activity. These gut-derived AMPs not only inhibit the growth of harmful microorganisms but also simultaneously promote the proliferation of beneficial microbes, modulate microbial composition, and maintain the balance of the gut microbiota, thereby contributing to intestinal health (48–50). This is crucial for preventing the occurrence of intestinal diseases.

In recent years, many important scientific research results have been achieved in the study of AMPs, especially the integrated computational method for mining AMPs. In this study, we mined multiple AMP sequences from FMT donor’s fecal samples through an improved integrated computational method, and verified through *in vitro* experiments that the mined AMPs have potent antibacterial activity and good safety. We explored the antibacterial mechanism of AMPs through molecular simulation, and the simulation results showed that cAMP573 may play an antibacterial role by binding to DNA gyrase 1KZN. At the same time, we found through further analysis that cAMP573 may be unique to the *G. qucibialis* species, and the sORF is located inside the *FBP-III* gene.

Traditional AMP mining research methods are costly, but efficient active AMP mining can be achieved through the integrated computational method of AI and multi-omics data. Ma et al. constructed an integrated computational method based on deep learning and multi-omics data for AMP mining, and 83% of the mined AMPs have effective antibacterial activity (12). A recent study systematically mined and analyzed 63,410 metagenomic data and 87,920 prokaryotic bacterial genomes from different regions of the world by integrating multiple computational methods, and constructed a large-scale AMP database AMPsphere (51). These works have greatly promoted the development of AMPs, but there are still many areas for improvement in the downstream analysis of AMPs. Therefore, in this study, we expanded and optimized the computational method of Yue Ma et al. First, in order to improve the positive rate of predicted AMPs, we cross-validated with Macrel through the third-party prediction tool iAMPCN and performed solubility analysis on the predicted AMP sequences. Secondly, we evaluated the structural stability of AMPs through molecular dynamics simulation. Finally, the antibacterial mechanism of AMPs was explored through molecular simulation docking technology (14).

The experimental results showed that the candidate AMP cAMP573 has good antibacterial activity against *S. aureus* and *E. coli*. We then explored the possible antibacterial mechanism of cAMP573 through protein simulation docking technology. DNA gyrase is an important topoisomerase in the DNA replication process of prokaryotes. Through docking simulation, we found that cAMP573 can produce stable binding with DNA gyrase 1KZN from *E. coli* and DNA gyrase 2XCT from *S. aureus*, and the binding between cAMP573 and *E. coli* 1KZN is stronger than that of *S. aureus*. It is worth mentioning that the AMP identified in a recent study also showed stronger binding ability to *E. coli* 1KZN (52). BamA is a protein related to membrane protein synthesis in Gram-negative bacteria (53), and studies have shown that this protein is associated with bacterial resistance (42). Recently, Li Yang et al. screened AMP sequences targeting BamA through molecular dynamics simulation and verified the activity of these AMPs through *in vitro* experiments (42). Our docking simulation results also showed that there is a meaningful binding between cAMP573 and BamA.

The active AMP cAMP573 we discovered in this study may be unique to the species *G. qucibialis*. *G. qucibialis* belongs to the genus Blast bacterium, and there are few studies on this species. A recent study on gastrointestinal symptoms in patients with RYGB surgery found that *G. qucibialis* may have a protective effect on gastrointestinal dysfunction in patients (54). In addition, our analysis results showed that the sORF encoding cAMP573 is located inside the fbp gene. Fructose-1,6-bisphosphatase (FBPase) in bacteria mainly includes class I FBPase, class II FBPase and class III FBPase, among which class III is mainly found in Firmicutes and has low similarity with the first two classes of enzymes (55). Concurrently, we also observed that the candidate AMP cAMP314 is predominantly distributed within specific regions of the genome of *P. massiliensis*. Notably, research conducted by Ren et al. (56) has found a positive correlation between *P. massiliensis* and the efficacy of FMT in treating ulcerative colitis. There are a large number of sORFs in bacterial genomes, and more and more studies have shown that the proteins encoded by sORFs (SEPs) have important biological functions (57). However, due to the particularity of SEPs themselves and the limitations of existing research methods, the functions of a large number of sORFs and their encoded proteins are still unknown (58). Our results show that integrated computational methods based on deep learning will be a powerful tool for studying these sORFs.

## Data Availability

Publicly available datasets were analyzed in this study. This data can be found at: the deep learning model used to predict antimicrobial peptides was obtained from https://github.com/mayuefine/c_AMPs-prediction. The Metaproteomic data were collected from https://www.ebi.ac.uk/pride PRIDE project IDs: PXD005780, PXD008870, PXD003907 and PXD000114. The metagenomic data used for correlation analysis were collected from https://www.ncbi.nlm.nih.gov/sra, project ID: PRJNA319574. The raw sequencing datasets presented in this article are not readily available because it contains human sensitive genetic information that may disclose the privacy and confidentiality of the participants. Requests to access these raw datasets should be directed to the corresponding author. The processed metagenomic data have been deposited in GitHub and are accessible via the permanent link: https://github.com/pointwei/FMT-MetagenomicData.

## Footnotes

^1^ https://github.com/s-andrews/FastQC
